# Application of KNN and ANN Metamodeling for RTM Filling Process Prediction

**DOI:** 10.3390/ma16186115

**Published:** 2023-09-07

**Authors:** Boon Xian Chai, Boris Eisenbart, Mostafa Nikzad, Bronwyn Fox, Ashley Blythe, Kyaw Hlaing Bwar, Jinze Wang, Yuntong Du, Sergey Shevtsov

**Affiliations:** 1 Faculty of Science, Engineering and Technology, Swinburne University of Technology, Hawthorn, VIC 3122, Australia; bchai@swin.edu.au (B.X.C.);; 2 CSIRO, Clayton, VIC 3168, Australia; 3 China Ship Scientific Research Center, Wuxi 214082, China; 4 Department of Transport, Composite Materials and Structures, Southern Center of Russian Academy of Science, 344006 Rostov-on-Don, Russia

**Keywords:** numerical analysis, process simulation, resin transfer molding (RTM), resin flow

## Abstract

Process simulation is frequently adopted to facilitate the optimization of the resin transfer molding process. However, it is computationally costly to simulate the multi-physical, multi-scale process, making it infeasible for applications involving huge datasets. In this study, the application of K-nearest neighbors and artificial neural network metamodels is proposed to build predictive surrogate models capable of relating the mold-filling process input-output correlations to assist mold designing. The input features considered are the resin injection location and resin viscosity. The corresponding output features investigated are the number of vents required and the resultant maximum injection pressure. Upon training, both investigated metamodels demonstrated desirable prediction accuracies, with a low prediction error range of 5.0% to 15.7% for KNN metamodels and 6.7% to 17.5% for ANN metamodels. The good prediction results convincingly indicate that metamodeling is a promising option for composite molding applications, with encouraging prospects for data-intensive applications such as process digital twinning.

## 1. Introduction

Fiber-reinforced composites, due to their exceptional mechanical properties while being lightweight, see ever-growing application and demand in various sectors such as aerospace and automotive [1,2,3]. The resin transfer molding (RTM) process has thus seen frequent adoption in the aforementioned industries due to its immense potential for cost-effective, high-volume composite production. RTM involves the distinctive manufacturing stages of preforming, mold filling, and part curing/demolding. In particular, the mold filling stage, where resin enters the closed mold via the injection gates to impregnate the dry reinforcement material within while air, volatiles, and excess resin escape the mold through the air vents, is vital to production efficiency and product quality. It is of paramount importance that the mold’s injection configuration be designed appropriately to ensure that a thorough impregnation of the reinforcement can be achieved within the shortest amount of time.

**Figure 1:**
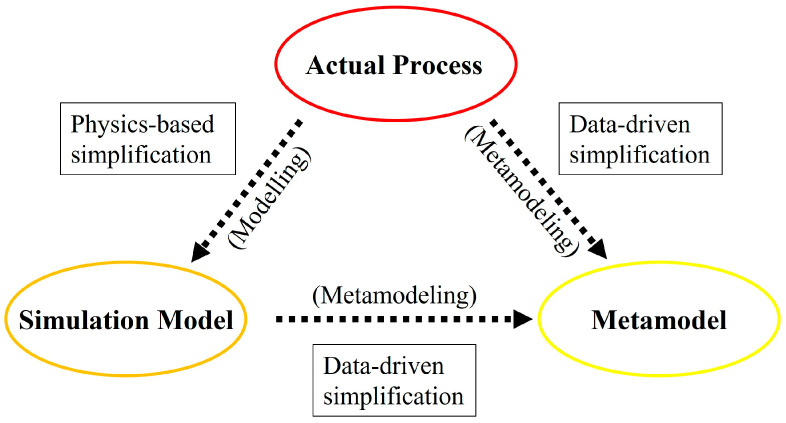
The derivation of various simplified models from the actual process.

The application of process simulation has immensely facilitated the demanding task of injection configuration optimization for RTM. However, currently, the multi-physical, multi-scale phenomenon of RTM mold filling requires a great deal of computational power to simulate at high resolution [4,5,6,7]. This calls for an alternative approach that can reduce the volume of process simulations required while still providing comparable results. To achieve the goal, the deployment of metamodeling approaches can be considered [4,6,8,9]. Metamodeling refers to the data-driven approach of constructing a simplified model of the process to represent the functional relationship between the manipulated input feature(s) and the resultant output feature(s) [6,9,10,11]. Unlike physics-based process simplification, metamodeling is a theory-agnostic (applicable in any field) approach where no prior knowledge of the process is required to establish correlations [10,11,12,13]. Figure 1 depicts the various approaches to process simplification and model derivation from the original, actual process. The simulation model is essentially an abstraction of the actual process, where only a selected subset of inputs is considered. The metamodel is then a further abstraction of the actual process or simulation model, where even fewer representative inputs are considered. At the expense of some process accuracy, metamodels are generally cheaper to execute when compared to the actual process or simulation model [8,9,11,14].

**Figure 2:**
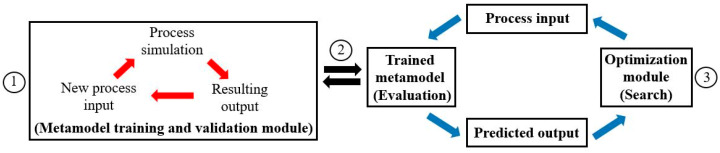
Schematic diagram of a metamodeling-integrated simulation-based optimization framework [9]. (This image was previously published in [9]).

The aim of metamodeling is to build predictive surrogate models that relate the input features with the output features based on training data attained computationally or experimentally to forecast the resultant output features when new input features are introduced. In the simulation-based optimization setting, the application of metamodeling effectively reduces the total number of simulation evaluations required, as the data needed for metamodeling is typically lower than that required throughout the entire simulation-based optimization process [4,8,9,11]. At the cost of some process accuracy, search and optimization can be performed more economically on the metamodel in place of the costly numerical simulation, as shown in Figure 2. Additionally, metamodeling can also provide valuable insights into the process’ underlying input–output relations [6,9,11]. The application of metamodeling in composite manufacturing opens up new avenues for real-time (online) process control/optimization and the development of digital process/material twins, which were previously restricted by the long computational time of numerical simulations [12,15,16].

As highlighted in recent reviews by Cassola et al. [5] and Mendikute et al. [4], the application of machine learning and metamodeling techniques for composite manufacturing is still in its early stages and requires more development. This is particularly true for the specific application of RTM injection configuration design and optimization, where contemporary applications are lacking or rudimentary [11,14,16,17]. Hence, in this paper, two metamodeling approaches, namely the K-nearest neighbors (KNN) metamodel and the artificial neural network (ANN) metamodel, are developed and investigated. The main contribution of this study lies in the performance investigation of these metamodels for the application of RTM injection configuration design. The feasibility of metamodeling to alleviate the computational burdens of simulation-based optimization is also assessed. In this study, aspects of metamodel development and parameter tuning are explored to provide valuable guidance for future applications.

This paper is structured as follows: Section 2 presents the detailed problem description and methodology to provide an overview of the problem domain, along with a quick linearity investigation to determine the nature of the datasets investigated to assist metamodel selection and development. In Section 3, the introduction and development of KNN metamodels are presented. In Section 4, the introduction and development of ANN metamodels are presented. Section 5 presents the results and discussions, followed by the conclusion in Section 6.

## 2. Problem Description and Methodology

**Figure 3:**
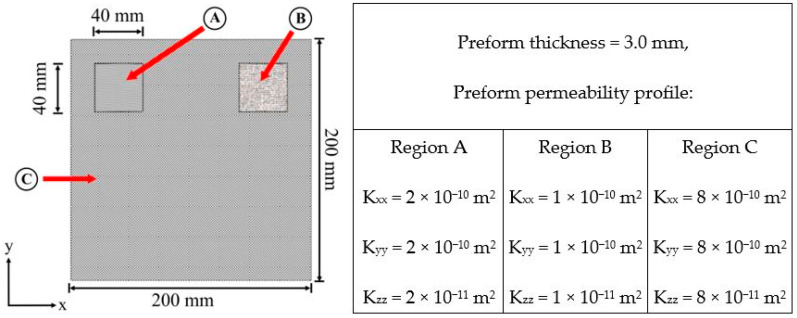
Dashboard panel [7]. (This image was previously published in [7]).

This study considers a single-gate resin transfer molding mold filling process for a dashboard panel part. Similar composite structures have been investigated in studies [7,13]. The composite part chosen is designed with a complex material profile made up of spatially inhomogeneous glass fiber reinforcements. To increase the non-linearity and complexity of the problem space, the fibrous reinforcements within the two notch areas are modeled to have different permeabilities from each other and from the main area (plate) of the preform. The material properties are presented in Figure 3. As observed from the part image, there are multitudinous options for where the resin injection gate could be placed on the surface of the mold. However, evaluating all the possible injection configurations enumeratively via numerical simulation would incur a massive computational cost. Hence, the objective of this study is to create predictive metamodels capable of relating the mold-filling process input–output correlations to assist mold configuration designing. The input features considered in this study are the (*x*, *y*) positioning of the resin injection gate and the resin viscosity.

**Figure 4:**
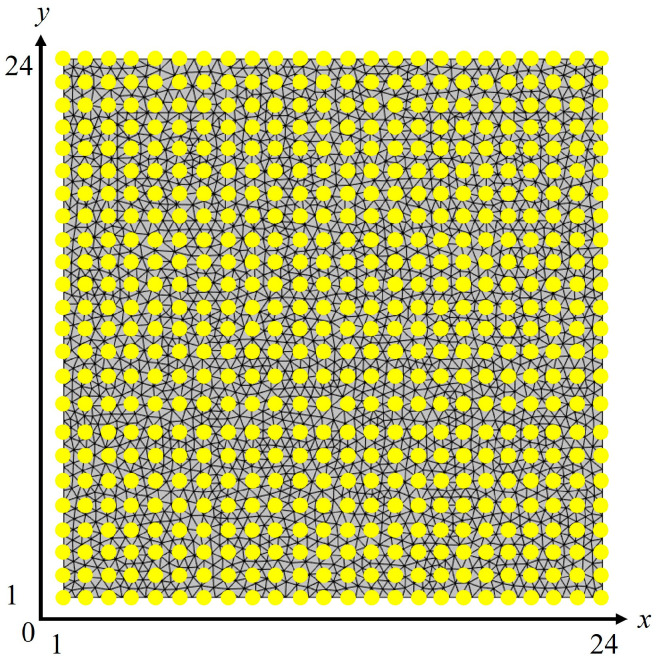
The 576 potential resin injection locations (yellow dots) on the mold surface projected on a (*x*, *y*) plane.

The first input feature investigated in this study is the (*x*, *y*) positioning of the resin injection gate. The top surface of the mold (i.e., top view) is first projected onto a two-dimensional Euclidean plane (*x*, *y*), with the *x*-axis representing the length of the mold and the *y*-axis representing the width. On the two-dimensional plane, 576 data points are formed uniformly on a 24 by 24 grid basis, as shown in Figure 4. For the single-gate injection process investigated, each data point (coordinate) represents a potential resin injection location (depicted as yellow dots), with the (*x*, *y*) input features ranging from position (1, 1) to (24, 24). The second input feature investigated is the resin viscosity. It is widely acknowledged in the literature that variations in the resin viscosity are common during RTM mold filling, typically arising from inconsistencies in its mixture ratio (e.g., with hardener, coloring, etc.) or variations in the resin/mold temperature [2,8,16]. It is thus of interest to create metamodels capable of predicting mold-filling performance at various viscosity levels. In-house experimental testing was performed to identify a suitable resin viscosity range for the metamodel’s development. Using a rotational viscometer, the Gurit low-viscosity epoxy Prime^TM^ 20LV was studied at 30 °C. Upon several repetitions, a resin viscosity range of 0.13 Pa·s to 0.23 Pa·s was obtained. Thus, for the second input feature, the resin viscosity was modeled at three instances: 0.13 Pa·s, 0.18 Pa·s (midpoint), and 0.23 Pa·s. The corresponding output features investigated are the resultant maximum injection pressure and the number of vents required. These two output features are chosen as they strongly determine the mold complexity and manufacturability, ultimately dictating the equipment cost [3,7,12].

In this problem context, the trained metamodel was tasked with predicting the resultant resin injection pressure and number of vents based on the given position of the resin injection gate (*x*, *y*) and resin viscosity. Cost savings can be attained by only simulating a portion of the data points to train the metamodel and utilizing it to predict the remainder. In this study, numerical simulations were performed to generate the datasets for metamodel training and validation. The numerical process analyses were performed using the commercial software Autodesk Moldflow^®^ Synergy 2019. A global edge length of 5 mm was adopted in this study, which was determined by the software’s automatic mesh sizing, diagnosis, and refinement calculations to be sufficiently discretized.

**Figure 5:**
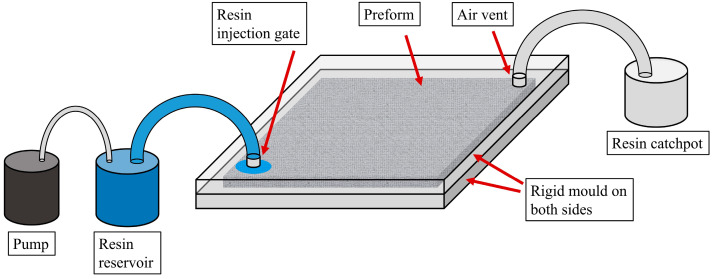
Schematic diagram of the experimental mold filling setup.

**Figure 6:**
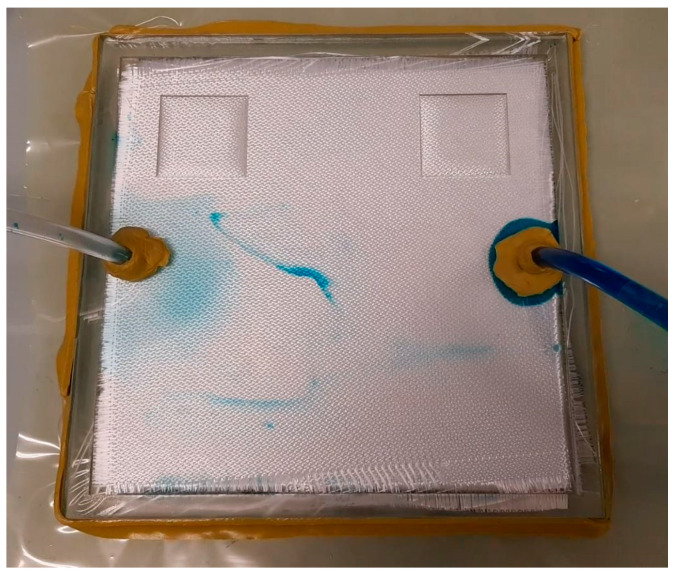
Image of the experimental setup (clear, rigid mold). Some stains are present on the exterior of the mold due to mold reusing, which does not affect the mold-filling process.

The process assumptions made in this study are similar to those in previous studies [3,7,17]. The single-gate, constant volume injection strategy is utilized in the mold filling simulations, with the injection time chosen to be 60 s. The mold-filling process is modeled to be isothermal with no occurrence of resin curing or race-tracking to simplify the process and minimize the simulation cost [3,7,9]. To ensure an accurate simulation of the mold-filling process, physical mold-filling experiments were performed to calibrate the simulation model. The schematic diagram and image of the mold filling setup are depicted in Figure 5 and Figure 6, respectively. Upon finetuning the simulation model empirically, the numerical simulation results are seen to closely match those of the experiments, which are deemed to be reasonably accurate and reliable (<5% discrepancy).

Upon optimizing the simulation model, data collection was carried out. To get a clear understanding of the underlying input–output relations, in this study, datasets were gathered with respect to each individual output feature. As there are two output features investigated in this study, separate data acquisitions were performed for each individual output feature.

For the first output feature, simulations were performed with varied injection locations to determine their resultant maximum injection pressure. The data collected from these simulations form the first dataset, which is named “Inj_XY”. Then, the simulations were repeated with varied injection locations and resin viscosity levels to determine the resultant maximum injection pressure for the second dataset, which is named “Inj_XYV”. Metamodels trained with these two datasets were tasked with predicting the resultant maximum injection pressure given an injection location and resin viscosity.

For the second output feature, simulations were performed with varied injection locations to determine the resultant number of vents required. The data collected from these simulations form the third dataset, which is named “Vent_XY”. The simulations were then repeated with varied injection locations and resin viscosity levels to determine the resultant number of vents required. However, it was manually identified (by human interpretation) that the number of vents in the dataset is invariant to the resin viscosity variations. Hence, it was not considered in this study. Metamodels trained with the “Vent_XY” dataset will be tasked with predicting the number of vents required by the given injection location.

In summary, three datasets “Inj_XY”, “Inj_XYV”, and “Vent_XY”, were collected to develop the metamodels. To reiterate, both input features *x* and *y* are whole numbers in the range of 1–24. Hence, the data from these two input features were declared an integer datatype. The other input feature (resin viscosity) is declared a double datatype as it contains decimals. As for the output features, the injection pressure data obtained contains decimals. Conversely, the number of vents dataset consists of whole numbers as the feature cannot be fractional. As a result, the data from the output features of maximum injection pressure and number of vents are declared as double and integer datatypes, respectively. The datasets investigated and their respective input/output features are tabulated in Table 1. The dissimilarity in data type and magnitude of the output features may lead to data imbalance and bias, further supporting the decision to separate the output features into individual datasets (and resultantly separate metamodels).

Metamodel training can then be initiated upon data collection. As costly simulations are required to generate the metamodel training and validation data, in real-world applications, it is desirable to construct the metamodels using minimal data. However, it is known that the insufficiency in training data volume will lead to inaccurate metamodel predictions [6,8,18]. While ill-representation of the actual process is unacceptable, performing excessive simulations for metamodel training counters its intended purpose of cost reduction. It is thus of research interest to investigate the cost-accuracy trade-off during metamodeling. Hence, in this study, the metamodels developed were trained at different dataset sizes to determine their resultant prediction accuracies. The data allocation strategy adopted in this study for metamodel training is the leave-some-out (LSO) cross-validation method [4,5,18,19]. In essence, the datasets were partitioned into sets of training data and validation data to ensure an unbiased metamodel accuracy evaluation. For each dataset, metamodel training and validation were performed across different data proportions. Firstly, the metamodels were trained with only 25% of the entire dataset, with the remainder of the data used to evaluate the resultant prediction accuracy. The procedure is then repeated with 50% and 75% dataset proportions for the metamodel training. The metamodels’ prediction performance for each dataset at different data proportions is compiled and discussed in Section 5. The uniform data sampling technique was adopted to ensure that the data were sampled in appropriate proportions and to prevent any unintended bias in data subsets during metamodel training [4,8,19].

In this study, the metamodel prediction error is quantified by the root mean square error (RMSE). RMSE is an accurate and reliable measure of fit/error (prediction differences) widely adopted in modeling and metamodeling applications. In these applications, RMSE is the quadratic mean of the differences between the predicted and observed values, obtained by square-rotting the average of squared errors:

(1) $$RMSE=\sqrt{\frac{\sum_{i=1}^{n}{\left(x_{i}-x_{i}^{\prime }\right)}^{2}}{n}}$$

where $x_{i}$ is the predicted value, $x_{i}^{\prime }$ is the observed value, and *n* is the number of data point comparisons. RMSE serves as a good indicator and standard for comparing the prediction accuracy of metamodels for any particular dataset, but not across multiple datasets as RMSE is scale-dependent. Fortunately, RMSE can be normalized to allow meaningful error rate comparisons between datasets of different scales. In this study, the RMSE is normalized by the range of the measured data to become the normalized root mean square error (NRMSE):

(2) $$NRMSE=\frac{RMSE}{y_{max}-y_{min}}$$

where $y_{max}$ is the largest measured value and $y_{min}$ is the smallest measured value. The computation of NRMSE allows for unbiased error rate comparisons across datasets, which is extremely valuable in this study as the two output features exist at different scales and magnitudes. With the measure of error defined, the hyperparameters of each metamodel can be tuned to minimize their prediction error. In the metamodeling context, the hyperparameters are parameters that modulate the metamodel’s learning process. The processes of hyperparameter selection and tuning are discussed in the metamodels’ respective sections.

**Figure 7:**
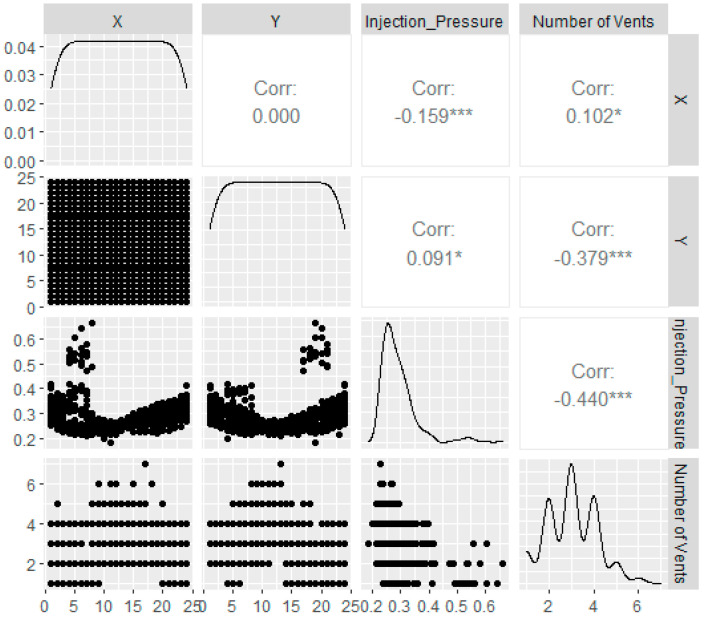
*ggpairs* plots depicting the various correlations between the input and output features.

Before developing the metamodels, it is of great importance to first identify the nature of the datasets investigated. This allows for the appropriate selection of metamodels to adopt with respect to the datasets in hand to prevent poor prediction performance and undesirable efficiency [6,11,13,20]. As most contemporary metamodels can be classified into the two broad categories of linear and non-linear models, an analysis is performed to investigate the linearity of the datasets in hand. It is known from inspection of the datasets in hand that the *x* and *y* positions of the resin injection gate strongly influence the resultant required injection pressure and the number of vents required. However, the nature of their correlation cannot be easily determined via human interpretation due to the huge number of data points present. Hence, the *ggpairs* function from the *GGally* package in software R (version 4.2.0 Vigorous Calisthenics) is employed to uncover the underlying variable correlations. The scatterplots, Pearson correlations, and density plots between the input features (*x*, *y* positions of the injection gate) and the output features (injection pressure, number of vents) are plotted in Figure 7. In the figure, the scatterplots are presented on the left side of the plot, the Pearson correlation values are presented on the right side of the plot, and the density plots are presented along the diagonal axis of the plot.

The Pearson correlation coefficient (PCC), or Pearson’s *r*, is investigated here as it is an effective measure of linear correlations between the variables [6,16,17]. As observed from Figure 7, no significant linear correlation can be observed between the input features and the output features. Thus, linear metamodels should be avoided in this study. The strong-to-medium negative correlation between the two output features suggests that there is an association between the two variables. This finding further supports the decision to create separate models for them to investigate the impact of the gate location variations on each of the response features individually [4,11,16,21]. Note that, in this variable significance investigation, the ‘resin viscosity’ variable is exempt. This is so because the ‘number of vents’ datasets remained consistent when the resin viscosity changed. This phenomenon implies that in the current mold filling configuration, the resin viscosity is not influential on the number of vents required. On the other hand, for the ‘injection pressure’ dataset, it is already widely established that the resin viscosity directly correlates to the resultant resin injection pressure in any mold-filling scenario [3,4,9]. Therefore, such an investigation is trivial.

## 3. K-Nearest Neighbors Metamodel

The first metamodel developed and investigated in this study is the K-nearest neighbors metamodel. KNN is a non-parametric supervised machine learning algorithm (lazy learning), broadly adopted for classification and regression problems [10,18,19,22]. KNN is a simple, easy-to-implement, yet powerful machine learning method that operates under the assumption that similar things are typically located in close proximity to each other. The reliability of KNN metamodeling thus hinges on this regularity being true and common enough for the model to be useful. The KNN algorithm predicts the label of the query point (dependent variable) corresponding to various training points (independent variables) by calculating the distance between the points on a feature space, which resembles the concept of similarity (i.e., proximity, closeness). During KNN metamodel development, there are two hyperparameters requiring decision-making and tuning: the type of distance metric to adopt and the value of *k*. There are numerous distance metrics available for the KNN distance computation, while the *k* parameter in the K-nearest neighbors metamodel refers to the number of nearest neighbors (of the query point) to consider.

To develop a KNN metamodel, the dataset to be analyzed is first split into two sets: a training dataset and a validation dataset. Next, the hyperparameter *k* is defined, and the distances of each (known) training data point with respect to the (unknown) query point are computed. The distances obtained (and their corresponding index) are then sorted into an ordered collection, arranged in ascending order by the magnitude of the distance. The first *k* entries from the sorted collection are then selected, with their labels identified for the purpose of prediction. For classification applications, KNN classifies the query point based on the majority of its nearest neighbors. For regression applications, KNN computes the mean of the nearest neighbors to predict the label for the query point. The distance computation and selection of neighbors take place when the query is made, with no explicit training step necessary. In some sense, *k* determines the size of the locally adaptive search window. Sparsely sampled data will result in a larger window, and the inverse is true. Operating on the principle of decision boundaries based on the *k* value, the distance to the *k*th nearest neighbors can also be seen as a local density (spatial) estimate.

### 3.1. Strengths and Drawbacks of the KNN Metamodeling Approach

The KNN metamodeling approach is widely adopted for several reasons. Firstly, KNN is a simple, intuitive, and interpretable algorithm as compared to most other contemporary machine learning algorithms. The mechanism simplicity of KNN eases the challenging tasks of model development and hyperparameter tuning, both of which typically require extensive expertise from the user to execute [20,23,24]. Additionally, there is minimal decision-making and hyperparameter tuning required of the user by the KNN metamodel. Besides that, the KNN algorithm is a versatile model that can be slightly modified to solve either classification or regression problems, depending on the problem requirements and dataset in hand. Being a non-parametric method, KNN does not require computing any prior information regarding the data distribution (unlike popular algorithms such as the Naïve Bayes method), making it easy to adopt and implement [20,22,24]. Furthermore, since no assumption of the data is made (e.g., functional form or shape), KNN has great flexibility and accuracy across both linear and non-linear datasets [20,24,25]. KNN’s sensitivity to the local structure of the data also allows it to perform well in datasets with noise or variabilities. Nevertheless, as no assumptions are made about the underlying data, no confidence in P(y∣x) can be guaranteed as a consequence of not computing any prior.

There are also drawbacks to the KNN metamodeling approach. The main drawback of KNN lies in its key requirement of computing all the distances between each data point present [20,25,26]. As a result, the time complexity of the KNN algorithm becomes *O*(*nd*), where *n* is the total number of data points in the training data and *d* is the total number of features in the dataset. Hence, when dealing with a huge dataset or with multitudinous features, KNN’s distance computations may lead to massive time complexity. KNN metamodels are thus not suitable for low-latency applications, as all computations are delayed until the query is made of the metamodel. Nonetheless, despite the drawbacks mentioned, KNN is chosen in this study as the KNN metamodel is widely proven to be reliable for solving numeric regression problems [20,22,25,26]. Most importantly, the datasets investigated in this study are not huge. The Inj_XY and Vent_XY datasets contain at most 576 rows of data points each, spanning across a two-dimensional feature space (i.e., number of features = 2). Meanwhile, the Inj_XYV dataset contains at most 1728 rows of data points spanning across a three-dimensional feature space (i.e., number of features = 3). For such small datasets, the computational cost of KNN metamodels is well-practicable [18,19,20,26].

### 3.2. Previous Work

The KNN metamodeling approach has often been adopted in the field of composite manufacturing to solve various classification and regression problems. Studies of particular interest and relevance adopting the KNN metamodeling approach in the literature are provided and briefly discussed here.

- Kessler and Rani [27] investigated the performance of various classification models (KNN, ANN, and decision tree algorithm) to predict the presence, type, and severity of damage in smart composite laminates. Using the damage-sensitive features from the Lamb wave response of composite laminates as predictors, the authors reported that the KNN metamodel performed best for this specific application.
- Maisarah et al. [23] utilized the KNN metamodel to classify a range of composite plates based on their natural frequencies and their corresponding amplitudes. The authors reported classification accuracies of more than 90%, showcasing the reliability of KNN given sufficient training data.
- Sharma et al. [19,26] developed a KNN metamodel to predict the fracture toughness of silica particulate-reinforced epoxy composites. Using the four material input parameters of aspect ratio, time, filler volume fraction, and elastic modulus, the KNN metamodel developed was able to predict the resultant material fracture toughness with an accuracy of 96%.
- Koumoulos et al. [18] created a KNN metamodel to identify and predict the unknown composite reinforcement type. The results demonstrate that undersampling greatly reduces the metamodel’s prediction accuracy, which the authors attribute to the increased risk of losing important information contained in the majority class.
- Ali et al. [10] attempted to create digital material twins based on the segmentation of two-dimensional micro-computed tomography images of the fibrous reinforcements with the aid of KNN and ANN. Two different reinforcement types were investigated, where one dataset contains an under-represented class (~5%) and another dataset has no class imbalance issues. The authors reported that ANN predicted more accurately than KNN in general, albeit both metamodels struggled in their predictions to identify the under-represented class due to the class imbalance.

To the author’s knowledge and best abilities, the KNN metamodeling approach has not been previously applied to the specific application of RTM mold filling process/mold configuration optimization, indicating the novelty and contribution of this study.

### 3.3. Distance Metric Selection and Hyperparameter Tuning for k

There are multiple distance computation metrics available to calculate the distances between the query point and training points within the dataset for KNN [22,23,25,26]. It is critical to note that the selection of which distance metric to adopt largely depends on the type, property, and dimensionality of the dataset. Additionally, the problem context also needs to be considered, as some metrics may be more appropriate for classification applications while others may be better suited for regression applications [4,22,25,26]. The datasets investigated in this study are of numerical values (non-negative, real value), lying on either a two-dimensional plane (*x*, *y*) or a three-dimensional plane (*x*, *y*, *v*). Hence, the Euclidean distance metric is adopted as the input features investigated are structured on low-dimensional planes (*2D*, *3D*), where the Euclidean distance is easy to compute and comprehend compared to the other distance metrics.

Since KNN metamodeling relies on distance computation for regression, it is typical that the scales (feature ranges) of the predictor features are normalized. This is because the features investigated may be represented in different physical units or unit scales, making the comparison convoluted and insignificant. However, in this study, no weighting or scaling is performed on the dataset (i.e., *k* is declared in a uniform weighting function form). This is so as both features investigated (*position in length and width*) were originally of the same measurement unit (*millimeter*, mm) and magnitude before being translated into Cartesian coordinates; hence, no bias or imbalance is present. Thus, within the KNN metamodeling developed in this study, with the mean of the nearest neighbors used for prediction, each data point is allowed to contribute unvaryingly and insensitively to their relative distance. Additionally, it is vital to highlight that the dataset investigated in this study exhibits a unique geometric structure where the distances between each data point are similar. Hence, in this study, the KNN metamodel is slightly modified to consider *k*-subsets of training points (of similar distance from the query point) rather than just *k*-individuals with the closest distance.

The next hyperparameter requiring tuning within the KNN metamodel is its *k* value. The KNN metamodel’s prediction performance is significantly dependent on the *k* value selected [18,22,25,26]. The *k* value determines how many nearest neighboring data points/distance subsets are considered during the metamodel prediction computation, which is entirely data-dependent [18,19,20,26]. Some points to consider when choosing the *k* value include the nature of the dataset, how sparsely the data points are located, and the presence of outliers and dataset imbalance. A small *k* value allows for detailed predictions but may lead to overfitting, while a large *k* value results in smoother predictions but may cause underfitting. As the optimal *k* value is dependent on the dataset and problem context, the general consensus for finding the optimal *k* value is trial-and-error and cross-validation [10,18,19,25]. There is, however, a well-known heuristic formula for finding a suitable *k* value, which states that the optimal *k* value should be around the square root of *n*:

(3) $$the\;k\approx \sqrt{n}$$

where *n* is the number of rows in the dataset (i.e., the number of data points). The dataset proportion selected for investigation is the 50% training proportion (of each respective dataset) for simplicity. The recommended *k* values for each dataset are presented in Table 2. Note that the recommended *k* values calculated are quite high, especially for the current datasets where the majority of the data are hidden from training. Using such high *k* values may result in underfitting. Thus, to identify the best *k* value to adopt for each dataset, trial-and-error investigations were performed.

**Figure 8:**
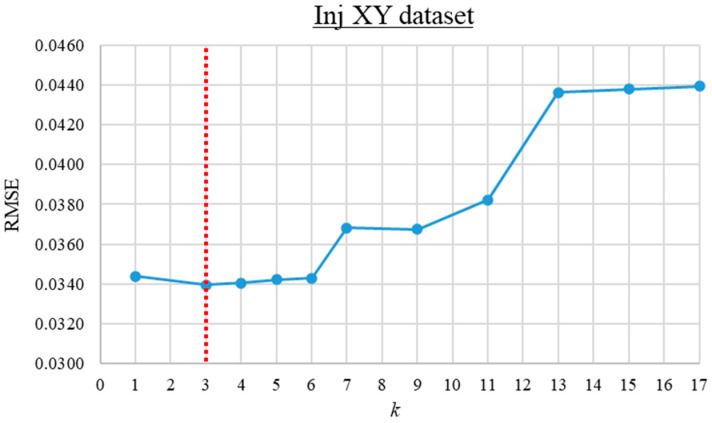
The root mean square error versus *k* value graph for the Inj_XY dataset.

**Figure 9:**
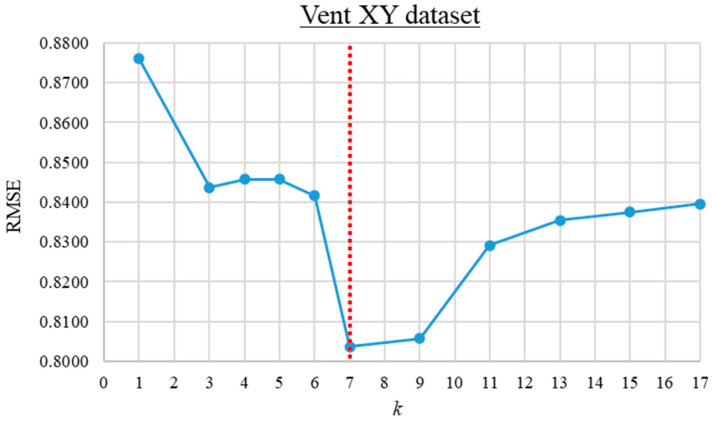
The root mean square error versus *k* value graph for the Vent_XY dataset.

**Figure 10:**
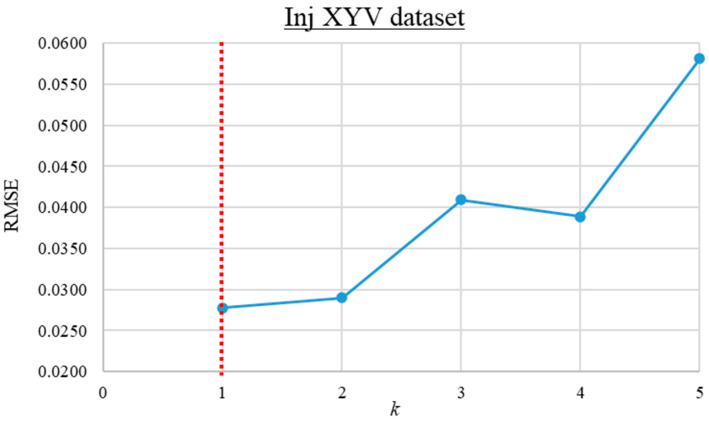
The root mean square error versus *k* value graph for the Inj_XYV dataset.

The trial-and-error search for the optimum *k* value was first performed for the Inj_XY and Vent_XY datasets. For these datasets, the RMSE of the KNN predictions versus the *k* value ranging from 1 to 17 is computed and presented in Figure 8 and Figure 9. As observed from the graphs, the prediction error is lowest when *k* is 3 and 7 for the Inj_XY and Vent_XY datasets, respectively. Based on insights gained from the previous *k* value investigations, the trial-and-error search for the optimal *k* value for the Inj_XYV dataset was initiated from 1 until no further improvement was obtained across four consecutive iterations. The RMSE of the KNN predictions versus the *k* values for the Inj_XYV dataset is plotted in Figure 10. Based on the graph presented, the prediction error is the lowest at *k* equal to 1. The good performance of low *k* values across the datasets investigated implies that the datasets in hand do not contain much noise. Therefore, these *k* values were used to build the other KNN metamodels (of each respective dataset) at different data proportions.

### 3.4. KNN Metamodel Development and Coding

In this study, the development and investigation of KNN metamodeling are focused on the application of regression. Note that due to the unique geometric structure of the datasets investigated, where the distances between each data point are similar, the KNN metamodels developed in this study are slightly modified to consider *k*-subsets of training points (of similar distance from the query point) rather than just *k*-individuals within the closest distance. The KNN metamodels investigated in this study are coded and developed in the software R. The KNN algorithm’s pseudocode is presented in Table 3. It is critical to highlight that, unlike most machine learning metamodels, KNN metamodels are not trained beforehand but rather run at the time of execution to find the output prediction. Hence, the KNN metamodels’ computational time is solely quantified by their execution time in this study.

## 4. Artificial Neural Network

**Figure 11:**
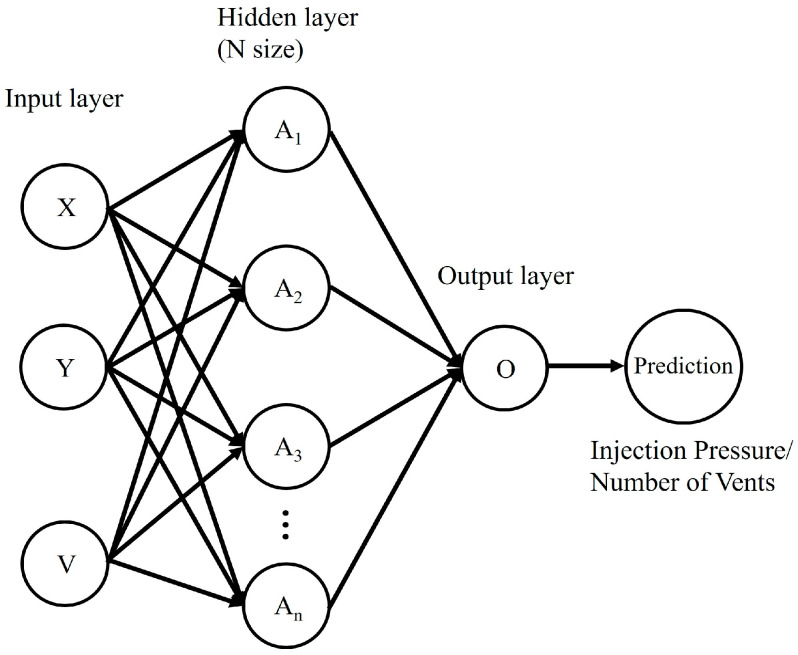
Schematic diagram of the ANN metamodel architecture developed and investigated in this study.

The second metamodel developed and investigated is the artificial neural network metamodel. ANN is a popular machine learning algorithm widely adopted for classification and regression problems [6,20,21,24]. ANN mimics the learning capabilities of the human brain, with interconnected nodes representing artificial neurons. Input data flows through the cascading layers of the input layer, hidden layer(s), and output layer, with each layer comprising nodes characterized by their weight, bias, and activation function. A schematic diagram of the metamodel architecture developed and investigated in this study is depicted in Figure 11. The number of nodes in the input layer is equivalent to the number of input features investigated. Within the hidden layers, the nodes receive the input data and perform certain operations based on the weights and biases, with the output then passing through an activation function before reaching the next layer. Most data processing within ANN metamodels is performed in the hidden neurons within the hidden layers. There can be any number of hidden layers within ANN metamodels. The cycle repeats if the next layer is also a hidden layer or if the data is transferred to the output layer to be presented as the predicted output features. The number of nodes in the output layer is dependent on the problem type. For regression problems such as those investigated in this study, the output layer will only have a single neuron.

Unlike KNN metamodels, ANN metamodels do not rely on prerequisite process regularities and can uncover the underlying relationships within the dataset through generalization given enough data [14,15,24,28]. To develop an ANN metamodel, the dataset to be analyzed is first split into two sets: a training dataset and a validation dataset. Next, the architecture of the artificial neural network is decided. There are various variants of ANN architectures, each specializing in some specific applications. Some notable examples include convolutional neural networks (image recognition) and recurrent neural networks (audio/temporal). In this study, the prominent multilayer perceptron ANN metamodeling architecture is utilized since the datasets in hand are numerical in nature. Then, activation functions for the nodes within the hidden and output layers are selected in accordance with the problem and dataset in hand. In this study, the prediction and learning mechanisms of forward propagation and back propagation are both employed. It is interesting to point out that the learning and prediction mechanisms of ANN metamodels are similar for both classification and regression applications.

### 4.1. Strengths and Drawbacks of the ANN Metamodeling Approach

The ANN metamodeling approach sees frequent application across various fields due to its outstanding merits in generalization and learning. Firstly, ANN metamodels have great adaptability and flexibility, capable of approximating both linear and non-linear relations [11,14,27,28]. Secondly, no prior knowledge of the dataset or the process/system that generates the dataset is required when developing the ANN metamodels, allowing for black-box applications [11,20,21,28]. Thirdly, the learning capability of ANN metamodels allows them to be trainable, which is a valuable trait, especially if a new, distinct occurrence/data arises. It also makes unobvious underlying features and relations indistinguishable by going directly from the inputs to the outputs to be extracted. Similar to KNN metamodels, ANN metamodels can also be employed for both classification and regression problems. On top of compressing the computation time required, the parallel architecture also brings a certain degree of fault tolerance, as the ANN metamodels will still be able to generate outputs if some of their nodes (computed by independent parallel machines) fail.

The ANN metamodeling approach is not without flaws. The main disadvantage of adopting ANN metamodels is their poor interpretability. ANN metamodels provide neither an explicit explanation nor logic for the symbolic meaning behind the learned/tuned model parameters [11,20,21,28]. Metamodels created via the ANN approach are fundamentally black boxes, hindering effective human interpretation and analysis. ANN metamodels’ parallel architecture is also a double-edged sword for their application; its parallel processing capability results in hardware dependence, requiring computing machines that support parallel processing in accordance with their structure. Furthermore, ANN metamodels require a huge training dataset to enable meaningful learning and accurate predictions [4,11,24,28]. Not only does this requirement impose restrictions on the nature of the dataset applicable, but it will also be computationally costly to compute and analyze the large dataset. There is also a lack of consensus on how ANN metamodels should be structured and tuned. While specific strategies are applicable in some specific cases, in most contemporary applications, ANN metamodels are generally structured and tuned through experience and strenuous trial and error. This poses enormous computational costs for the development and finetuning of ANN metamodels, which need to be compensated by their cost-saving capability to justify their adoption.

### 4.2. Previous Work

The ANN metamodeling approach has been frequently adopted in the field of composite manufacturing to solve a wide range of classification and regression problems. Note that in this section, only studies relating to the specific application of ANN metamodels for RTM mold-filling process control and optimization are included and discussed.

- Spoerre et al. [16] formed a simulation-based optimization framework by integrating the ANN metamodel with the genetic algorithm to optimize mold filling for the RTM process. Upon training the ANN metamodel with experimental data, GA was employed on the trained metamodel to search for the optimum process parameters. However, model tuning was not performed via automated weights/biases updates, but rather relied on the progressive manual addition of hidden nodes, which was proven to be costly and inefficient.
- Nielsen and Pitchumani [15] proposed an ANN metamodel-integrated online process control system for the RTM mold-filling stage. The ANN metamodel was trained with various mold-filling instances generated from numerical simulations, along with their resultant mold-filling performance (quantified by the volume of dry spots formed). When deployed, the active control system monitors the mold filling progression using the online sensors, with the information periodically fed to the trained metamodel to determine whether dry spots will form and if corrective actions are needed.
- Luo et al. [14] developed an ANN-GA optimization framework to optimize a single-gate RTM configuration with respect to filling time and dry spot formation. A rapid RTM process metamodel was created by training ANN through feedforward and back propagation strategies using simulation data. Upon metamodel training, the authors reported impressively low prediction errors of less than 6.3%. Nonetheless, their mold-filling setup was relatively simple and straightforward, slightly discounting the metamodel’s excellent performance.
- Okabe et al. [17] employed the self-organizing map (SOM) approach to uncover the implicit relations between the output features for a multi-objective RTM process optimization study. The SOM approach, which is a specific subtype of ANN, utilized unsupervised competitive learning to produce a topology-preserving map of the data to visualize the Pareto-optimal solutions obtained. The authors reported interesting relationships between the investigated processes’ output features. Note that SOM was utilized in this study for data mining and classification purposes rather than for regression or prediction purposes.
- Matsuzaki et al. [29] constructed an ANN metamodel to predict the resin impregnation time for the VA-RTM process. Cost savings were innovatively achieved by training the metamodel using 3D analysis data to predict the impregnation progression along the preform thickness when fed with 2D analysis data. However, the prediction accuracy is more reliant on the process regularity that the distribution media will be filled rapidly prior to impregnation along the thickness direction (for the specific part investigated) than the metamodel performance. On a simple flat-plate model, an average prediction error of 6.31% was reported.

As presented, to the authors’ knowledge and best abilities, the ANN metamodeling approach has not been previously employed for the prediction and optimization of the injection configuration for RTM mold filling. This research gap renders the current study novel and valuable to the advancement of contemporary composite molding capabilities.

### 4.3. ANN Metamodeling Procedure

To develop an ANN metamodel, the dataset is first split into two sets: a training dataset and a validation dataset. Then, the ANN architecture is decided. An overly simple ANN architecture with too few connections will result in a lack of capacity to extract the underlying regularities in the training data. Conversely, an excessively complex ANN architecture will result in overfitting where the ANN metamodel conforms to the training data too well and lacks generalization for unknown data outside of the training set. Interestingly, having more hidden layers also does not always equate to better predictions, yet the increase in computational cost is guaranteed [10,11,21,24]. There are no concrete guidelines for deciding how many hidden layers to adopt. For simplicity and computational efficiency, in this study, the ANN metamodels are constructed with one input layer, one hidden layer, and one output layer. This fundamental ANN architecture has been frequently adopted in related studies [6,11,15,24]. The adoption of such a non-complex architecture is mainly driven by the small dataset sizes and the application of back propagation learning in this study. Based on the universal approximation theorem, an ANN metamodel containing one hidden layer encompassing a finite number of hidden neurons is sufficient to approximate any continuous function [5,10,11,28].

The number of nodes in the input layer is set to be equal to the number of input features investigated, which is two for the Inj_XY and Vent_XY datasets and three for the Inj_XYV dataset. As the problem investigated is one of regression, the output layer will only have a single neuron. The schematic diagram of the ANN metamodels developed and investigated in this study is previously presented in Figure 11. After deciding on an ANN architecture, activation functions for the hidden and output layers are chosen and implemented, as discussed in the next section.

Input data passes through the artificial neural network framework via the forward propagation mechanism, where input data enters from the input layer and gets processed in the hidden and output layers, with the output then presented to the users. Details of the data processing progression are provided as follows:

To ease understanding, let *x^i^* be the sample input data, where (*i*) refers to the row number, the hidden layer denoted as the [1]st layer, and the output layer is denoted as the [2]nd layer. Input data received at the input layer is first passed on to the hidden layer without any modification. Then, in the hidden layer, a weight (*w*) and a bias (*b*) are introduced to the input to produce an output *Z* as follows:

(4) $$Z^{\left[1](i\right)}=w^{1}x^{\left(i\right)}+b^{\left[1](i\right)}$$

where the weight is the coefficient of the input, while the bias is a constant added to the product. Then, the output is passed through the hidden layer’s activation function, which is chosen to be the tanh function in this study (explained in the next section), as follows:

(5) $$a^{\left[1](i\right)}=\tanh\left(Z^{\left[1](i\right)}\right)$$

The output of the hidden layer, $a^{\left[1\right]}$, will then be passed on to the output layer, where another set of weights and biases are introduced again:

(6) $$Z^{\left[2](i\right)}=w^{2}a^{\left[1](i\right)}+b^{\left[2](i\right)}$$

Finally, the result obtained will go through the output layer’s activation function, which is chosen to be the sigmoid function in this study (explained in the next section), to produce a prediction:

(7) $$y_{prediction}^{\left(i\right)}=a^{\left[2](i\right)}=\sigma \left(Z^{\left[2](i\right)}\right)$$

The weights and biases of nodes within the hidden and output layers are initialized randomly, which can also vary across different nodes. Upon completion of feed-forward, the back propagation learning mechanism is applied in this study to improve prediction accuracy. The machine learning procedure begins with the calculation of the loss (i.e., error) between the predicted output and the known actual output (i.e., ground truth from the training data). The loss function is then differentiated to obtain the gradient of the loss, which is used as a basis to iteratively tune the weights and biases to minimize the loss [6,21,24]. In other words, the weights and biases are machine-tuned values that dictate the prediction accuracy. The updating of the weights and biases is controlled by a learning rate (α), as follows:

(8) $$w_{new}^{\left[i\right]}=w_{old}^{\left[i\right]}-\alpha \times dw^{\left[i\right]}$$

(9) $$b_{new}^{\left[i\right]}=b_{old}^{\left[i\right]}-\alpha \times db^{\left[i\right]}$$

The learning process is iteratively repeated until the epoch is reached, which is the predefined number of learning iterations. It is apparent that effective machine learning relies on the cooperation of the forward and back propagation mechanisms. During the ANN metamodel training, once the model parameters are initialized, forward propagation is performed to obtain the loss function, which is then used by back propagation to update the model parameters to be evaluated again.

### 4.4. Activation Function Selection

Within a neural network, the activation functions used within the nodes in the hidden layers and output layer are responsible for determining whether a neuron gets activated or not, ultimately determining the metamodel’s performance [6,14,24,28]. These mathematical functions act as mathematical gates, transforming the summed weighted inputs and biases from the node into output values for the next layer for further computation or output. The main aim of employing the activation functions in the nodes is to introduce non-linearities into the metamodel, allowing the model to approximate non-linear relations from the input features to the output labels [11,15,24]. This is essential, as the other operations within the neural network are linear in nature. 

It is critical to first highlight that it is advisable to use the same activation function across all the nodes within the hidden layers and a different activation function for that of the output layer [15,24,28]. This configuration allows for more adaptability. In this study, the sigmoid function and tanh function are selected as activation functions for the ANN metamodels developed. These two functions are deemed suitable as the dataset investigated is to be normalized into the range of 0 to 1. The Tanh function is chosen to be the activation function for nodes within the hidden layer due to its zero-centric symmetric nature, which eases back propagation learning. On the other hand, the sigmoid function is selected as the activation function for the output layer thanks to its output range of 0 to 1, which corresponds to the expected output range in this study. The potential negative output of the Tanh function makes it unsuitable for the output layer.

Note that the Vent_XY dataset contains output data ranging from 1 to 7, which can be greater than 1. Hence, data pre-processing is necessary. The output feature of the Vent_XY dataset is scaled down to the range of 0 to 1 by the following scaling formula:

(10) $$y^{\prime }=\frac{y-y_{min}}{y_{max}-y_{min}}$$

where $y^{\prime }$ represents the scaled value, y represents the original unscaled value, $y_{max}$ represents the maximum value within the dataset, and $y_{min}$ represents the minimum value within the dataset. This data pre-processing only applies to the Vent_XY dataset for the ANN metamodels.

### 4.5. Hyperparameter Tuning for the Number of Hidden Neurons, Epoch, Learning Rate

Next, the hyperparameters of the epoch, learning rate, and number of hidden neurons are tuned. Note that the momentum parameter commonly adopted in back propagation learning is not considered in this study as only a single hidden layer is employed. These hyperparameters are crucial to the effectiveness of back propagation learning [20,24,28]. However, the optimal magnitudes of these hyperparameters are dependent on the dataset investigated (complexity, outliers, and noise), which will vary on a case-by-case basis. There is a general consensus in the empirical literature that the tuning of these hyperparameters rests on extensive trial-and-error [6,11,14,28]. In this study, the empirical determination of said hyperparameters was aided by the thoughtful design of experiments (DoE) guided by knowledge gained from related literature studies.

The epoch refers to the number of learning iterations (i.e., each comprises one forward propagation and one back propagation). While having a high epoch will increase the likelihood of securing a good result, its increment will inflate the training time and may also result in overfitting. On the other hand, an excessively low epoch may result in underfitting as insufficient iterations are provided to the metamodel to discover the underlying data relations. Epochs are typically defined in the range of tens of thousands to ensure a sufficient learning period. Apropos of the relevant literature, in this study, the epoch is fixed at 100,000 [10,11,14,28]. This decision, guided by literature, was made because it is infeasible to optimize the epoch value via trial-and-error. Additionally, fixing the epoch in this study allows the other two learning parameters to be optimized accordingly.

The learning rate, shown previously in Equations (8) and (9), regulates the machine learning rate by controlling the magnitude of change of the weights and biases during back propagation. The learning rate should be a positive value ranging non-inclusively between 0 and 1. While a higher learning rate will lead to quick solution convergence, it may also cause solution overshooting and unstable training. Conversely, a smaller learning rate will result in a long training time or even failure to learn. The non-consideration of the momentum parameter makes the learning rate tuning extremely critical in this study. Hence, with respect to related studies from the literature, the learning rate is enumeratively tested across the arithmetic sequence of (0.1, 0.3, 0.5, 0.7, 0.9) in this study to identify the most suitable value for the hyperparameter [10,11,14,28].

Lastly, the hidden neurons refer to the neurons present within the hidden layer. The hidden neurons determine the fit of the model, allowing the metamodel to generalize to the dataset. A delicate tuning of the number of hidden neurons is of paramount importance, as an excessive amount will lead to overfitting, whereas an insufficiency in it can cause underfitting. It is also worth mentioning that the number of hidden neurons also affects the resultant computation time required. According to the relevant literature, the suitable number of hidden neurons for problems with small dataset sizes typically ranges between 5 and 25 [11,14,15]. Hence, the hyperparameter is iteratively tested from 5, with an increment of 5, until 25.

For the hyperparameter tuning experiments, the Inj_XY (50% data proportion) dataset was used as the baseline dataset to investigate the corresponding prediction performance. The epoch is fixed at 100,000, with the learning rate and the number of hidden neurons varied for tuning purposes. As the epoch is fixed at 100,000, the entire dataset was iteratively processed 100,000 times with the ANN model. The model is optimized after every iteration, and the result of the final optimized model was used for this study. The resultant prediction errors, in terms of RMSE, of the ANN metamodel at various parameter levels are compiled and presented in Table 4.

It is observed that the ANN prediction error is the lowest at a learning rate of 0.9, adopting 25 hidden neurons. As the results suggest that a further increase in the number of hidden neurons may improve the prediction accuracy, a further trial-and-error study was conducted up to a maximum of 50 hidden neurons. Further investigation of the learning rate is unnecessary, as observed from the results obtained. The resultant prediction errors, in terms of RMSE, of the ANN metamodel adopting different numbers of hidden neurons are compiled and presented in Table 5.

The results presented show no improvement in prediction accuracy as the number of hidden neurons increased past 25. Therefore, the optimum hyperparameters obtained empirically in this study are 100,000 for the epoch, 0.9 for the learning rate, and 25 for the number of hidden neurons. These hyperparameters were used to build the other ANN metamodels (of each respective dataset) at different data proportions. As the output data of the Vent_XY dataset has been normalized and exists in a similar magnitude as the injection pressure datasets, the issue of an *exploding gradient* is negated.

### 4.6. ANN Metamodel Development and Coding

In this study, the development and investigation of ANN metamodeling are focused on the application of regression. The ANN metamodels investigated in this study are coded and developed in the software R. The ANN algorithm’s pseudocode is presented in Table 6. Note that ANN metamodels require training prior to producing accurate predictions. Hence, in this study, the artificial neural network’s computational time is unbiasedly quantified by the sum of its training time and execution time.

## 5. Results and Discussion

**Figure 12:**
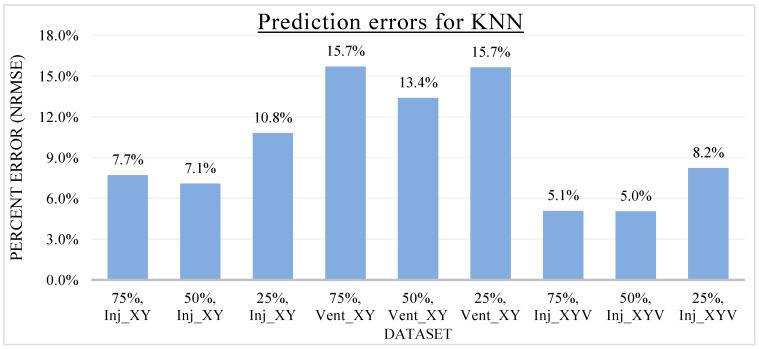
Prediction errors (NRMSE) of KNN for various datasets and data proportions.

**Figure 13:**
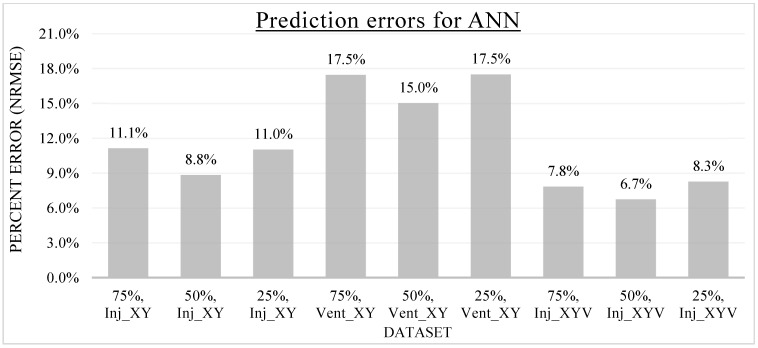
Prediction errors (NRMSE) of ANN for various datasets and data proportions.

In the previous sections, the specifics of KNN and ANN metamodels were discussed. After developing and training the metamodels, the metamodels were utilized to produce output predictions for the Inj_XY, Vent_XY, and Inj_XYV datasets. To investigate the effect of dataset sizes on the metamodel prediction accuracy, the investigated metamodels were developed using different training data proportions. The generalizability of the associated hyperparameters of the KNN and ANN metamodels is also investigated by the training data proportion variations. As the hyperparameters of both the KNN and ANN metamodels were tuned with respect to a specific training data proportion (i.e., 50% in this study), it is of research interest to evaluate the effectiveness of these hyperparameters across different training scenarios. The training data proportions are labeled as N%, where N% indicates the percentage volume of the original dataset used for the metamodel training, whereas the remaining (100-N)% of the dataset was used as the validation dataset to evaluate the metamodels’ prediction accuracy. The metamodel prediction errors were averaged with respect to the number of data points to allow for unbiased comparisons between models with varied validation data volumes. The metamodel prediction error is quantified in this study by the NRMSE introduced earlier. As the measure of error is normalized, percentage error comparison across distinct datasets is possible and reliable. The metamodel prediction errors for each dataset at various data proportions of the KNN metamodel and ANN metamodel are presented in Figure 12 and Figure 13, respectively.

Overall, the metamodel predictions were found to be quite accurate for both KNN and ANN metamodels. The percentage prediction error of the KNN metamodel ranges from 5.0% to 15.7%, while that of the ANN metamodel ranges from 6.7% to 17.5%. It is interesting to point out that the highest prediction error for both metamodels occurred for the Vent_XY dataset. The prediction errors for the Vent_XY dataset are consistently greater than the other datasets across different data proportions for both metamodels. This phenomenon could be attributed to the integer nature of the dataset (i.e., a whole number), which makes the data discrete. As the possible number of vents ranges discretely from one to seven, the lowest possible prediction error is already 1/7 of the total range, which is roughly equivalent to 14.3% in normalized error. The other datasets dealing with the resultant injection pressure (Inj_XY, Inj_XYV) do not face such an issue as their data consists of continuous decimal numerals. A possible solution to increasing the prediction accuracy for the Vent_XY dataset is to structure the problem into a classification problem and develop the metamodels accordingly.

Looking at the prediction errors of KNN presented in Figure 12, across all three datasets, the lowest prediction error occurred invariably at the 50% training data proportion. While it is logical that the metamodel prediction accuracy will increase when more training data is available, the increase in prediction accuracy is observed for the training data increment from 25% to 50% but not from 50% to 75%. This is likely due to the fact that the *k* hyperparameter was tuned for the investigated datasets at 50% training data proportion in this study. These results indicate that the hyperparameter *k* for KNN metamodeling may lack generalizability, although the reduction in prediction accuracy is not significant. When adopting the KNN metamodeling approach for a new dataset, it is thus advisable to tune the hyperparameter anew instead of relying on prior information or knowledge. Fortunately, the *k* hyperparameter is the only hyperparameter requiring tuning for the KNN metamodel. Consequently, the cost and difficulty of developing optimized KNN metamodels from scratch are not prohibitive.

A similar trend is observed for the prediction errors of ANN, as seen in Figure 13. Akin to the KNN metamodel development, the ANN metamodels were also developed and tuned in this study using the 50% training data proportion as the baseline. As a result, the ANN metamodels developed excelled in predicting the output features for all three datasets at the said data proportion. Strangely, while it is widely acknowledged that the increase in training data volume will improve the prediction accuracy for ANN metamodels [6,10,11,28], in this study, this behavior is inconsistent. In fact, the increase in training data proportion from 25% to 75% in this study produces minimal improvements to the prediction accuracy for all three datasets investigated, unlike that of KNN. This outcome infers that the hyperparameters of ANN exhibit lesser generalizability compared to those of KNN. As there are more hyperparameters to tune for ANN than KNN, this is expected. Nonetheless, the metamodel development and training for ANN are significantly more complicated and time-consuming than those for KNN.

It is also critical to highlight that, for both the KNN and ANN metamodels, the overall metamodel prediction error is the lowest for the Inj_XYV dataset. This is likely attributed to the increase in the number of input features investigated, where Inj_XYV consists of three input features while the others only have two. As an additional feature is added, the total volume of data will increase as well, leading to an increase in training data volume and, thus, more opportunities to discover the underlying relationships [11,12,21,24]. In addition, the successful development of the KNN metamodels on a three-dimensional feature space (*x*, *y*, *v*) demonstrates the suitability of adopting the Euclidean distance as the distance metric for low-dimensional problems. Nevertheless, as more input features are included, the dimensionality of the feature space will increase as well.

To ease the result analysis, the point-to-point comparison between the real and metamodel predicted values for each dataset and each metamodel at different training data proportions is graphed. The point-to-point comparisons for KNN across different datasets and data proportions are presented in Figure 14, Figure 15, Figure 16, Figure 17, Figure 18, Figure 19, Figure 20, Figure 21 and Figure 22. The point-to-point comparisons for ANN across different datasets and data proportions are presented in Figure 23, Figure 24, Figure 25, Figure 26, Figure 27, Figure 28, Figure 29, Figure 30 and Figure 31.

Overall, the KNN metamodels demonstrated slightly better prediction accuracy than the ANN metamodels across all investigations. Improvements in prediction accuracy of up to 3.4% can be attained when switching from ANN to KNN. Nevertheless, while the superior prediction accuracy of KNN is attractive, there are several other factors to consider when selecting the metamodeling approach to adopt. Some factors include the ease of interpretability, the volume of training data required, and the ease of development/implementation. Based on literature analyses, a qualitative comparison of the barriers to adoption between KNN and ANN is provided in Table 7.

Lastly, the computational costs (in terms of wall-clock time) for process evaluation via numerical simulation versus that of metamodeling (KNN, ANN) are compared. Table 8 tabulates the average computing time of the metamodeling approaches. As mentioned previously, KNN metamodels’ computation time is quantified solely by their execution time (as no training is required), while ANN metamodels’ computation time is quantified by the sum of their training time and execution time. Note that most of the computation time of ANN actually arises from training, as the execution time of the ANN metamodels developed is almost instantaneous (~1–2 s). On the other hand, each numerical process simulation performed using Moldflow requires an average of 729 s to compute. All computations were performed on a machine equipped with a 256 GB SSD, 8 GB of RAM, and a Ryzen 7, 4000 series processor.

When adopting the ANN metamodeling approach, high levels of computational time and user expertise are required for effective ANN development. Nonetheless, once the ANN metamodel is tuned, near-instant execution is greatly desirable, especially when multiple predictions are to be performed. In such a scenario, the long execution time of KNN is undesirable, as prior training cannot be performed due to the nature of the approach. As the dataset size increases, KNN will face the curse of dimensionality, while ANN’s computational load will not be as severely impacted as only the one-time training time is extended but not the execution time. Last but not least, it is worth highlighting that ANN metamodels possess the ability to learn and improve when provided with more training data and time, whereas KNN metamodels’ performance improvement is greatly restricted by their lazy learning nature [4,24,28].

## 6. Conclusions

The adoption of metamodeling in composite molding applications aims to alleviate the computational burdens of simulation-based optimization while maintaining credible process accuracy. In this paper, the application of KNN metamodeling and ANN metamodeling approaches to predict key mold-filling output parameters for the RTM process is presented. Multiple metamodels were created using different datasets and varied training data proportions to investigate the prediction performances of these metamodels. Both investigated metamodels demonstrated desirable prediction accuracies, with a low prediction error range of 5.0% to 15.7% for KNN and 6.7% to 17.5% for ANN. The good performances of the metamodels developed indicate that metamodeling is a promising option for minimizing the cost of optimization for composite molding applications. The results obtained do infer that the hyperparameters may lack generalizability, although the reduction in prediction accuracy is not significant. By virtue of the complex dynamics and interactions among the multitudinous correlative constituents during mold filling, metamodels developed are likely to be case-specific and lack generalization, meaning that a new metamodel may need to be constructed whenever a substantial change in any key parameter(s) occurs. Further investigations are necessary to verify and address the aforementioned conundrums.

This study could be seen as one of the early attempts to investigate the performance of metamodeling for the prediction and optimization of the injection configuration for RTM mold filling. The objective of developing metamodels capable of accurately predicting some output features given the input features is achieved in this study. In fact, the metamodel prediction accuracy is quite competitive compared to the simulation itself. Nonetheless, the prediction accuracy of the metamodels largely depends on the regularity of the process investigated. Note that the results and knowledge inferred from this study should be interpreted in light of its limitations. In particular, all of these studies dealt with data generated from deterministic simulations. The common stochastic phenomenon of race-tracking is negated in this study, which could slightly undermine the effectiveness of the optimal injection configuration obtained. The research advancement towards metamodeling the stochastic behavior of race-tracking is much needed and is planned as future work. In addition to stochastic process optimization, the application of metamodeling for online process optimization applications is also very attractive and promising. In addition, the approaches to metamodel development adopted in this study could be improved. Certain aspects, such as the ANN architecture or some metamodel parameters, were largely decided based on experience rather than actual experimentation in this study. The exhaustive evaluation of other metamodel development strategies or the focused research emphasis on metamodel developments could further enhance their prediction performance and efficiency.

In conclusion, this study demonstrates the promising potential for the adoption of metamodeling in composite molding applications. The hastened solution evaluation time brought by metamodeling can help further advance and develop future digital twining technologies, real-time and online process monitoring/optimization, stochastic process optimization, and much more. These technological advancements will alleviate contemporary composite manufacturing capabilities to a greater height, paving the way toward smart manufacturing-focused Industry 4.0. The metamodeling investigations conducted in this study will contribute to promoting and progressing the adoption of metamodeling in RTM mold configuration optimization applications, which is currently in its infancy, as seen from the lack of adoption in the literature.

## Figures and Tables

**Figure 14:**
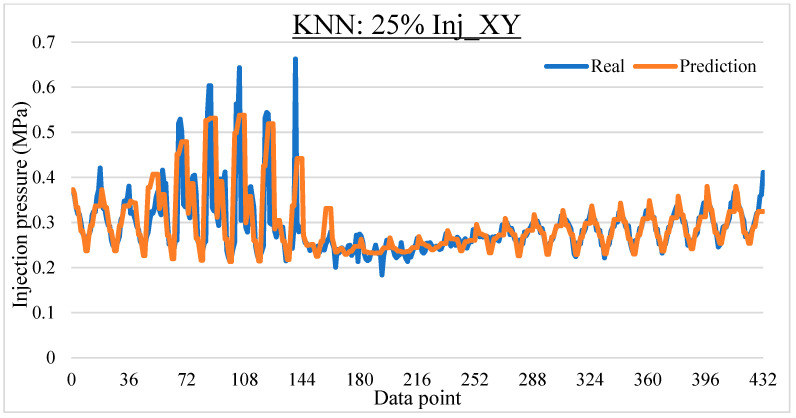
Comparison between the real and KNN predicted values for Inj_XY dataset at 25% training data proportion.

**Figure 15:**
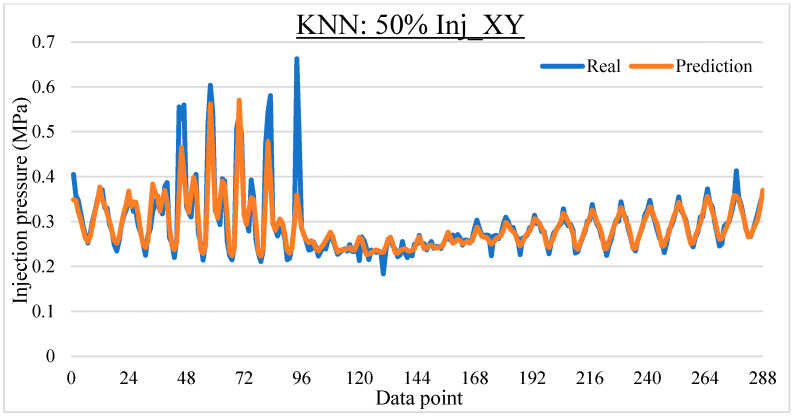
Comparison between the real and KNN predicted values for Inj_XY dataset at 50% training data proportion.

**Figure 16:**
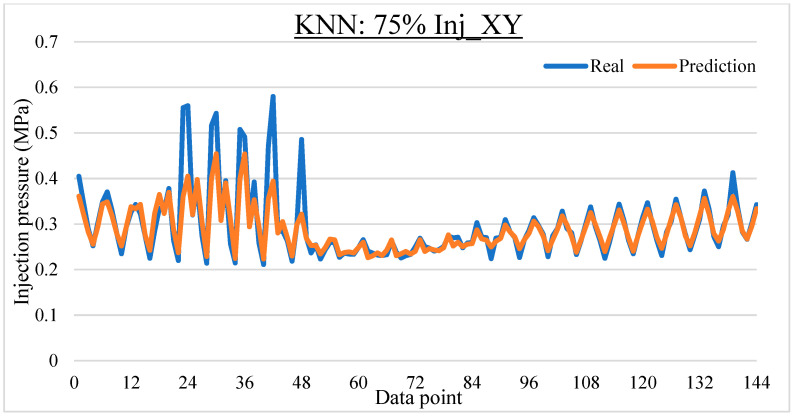
Comparison between the real and KNN predicted values for Inj_XY dataset at 75% training data proportion.

**Figure 17:**
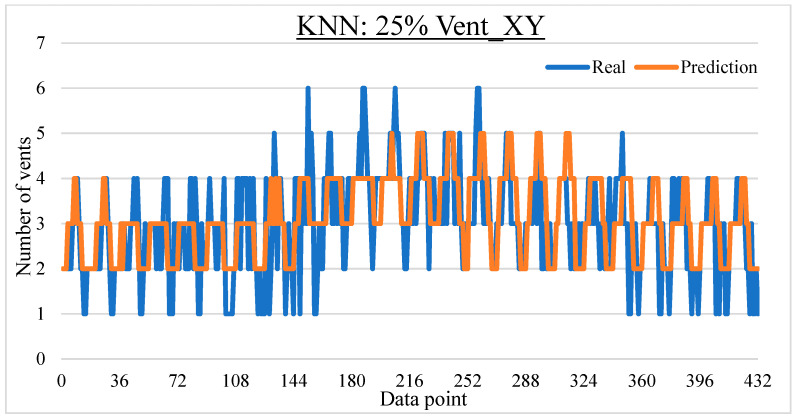
Comparison between the real and KNN predicted values for the Vent_XY dataset at 25% training data proportion.

**Figure 18:**
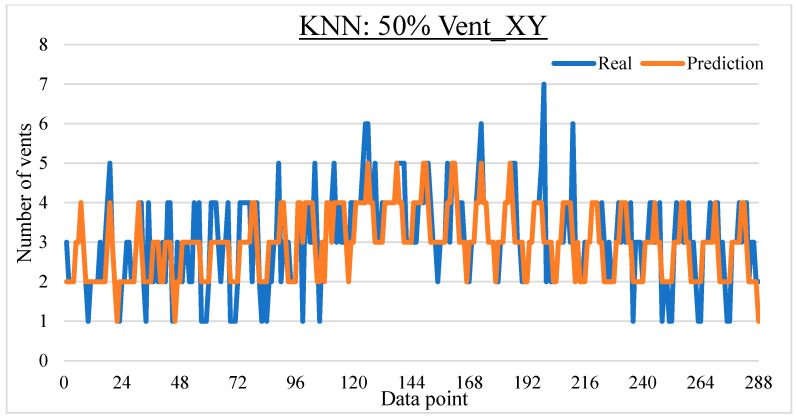
Comparison between the real and KNN predicted values for the Vent_XY dataset at 50% training data proportion.

**Figure 19:**
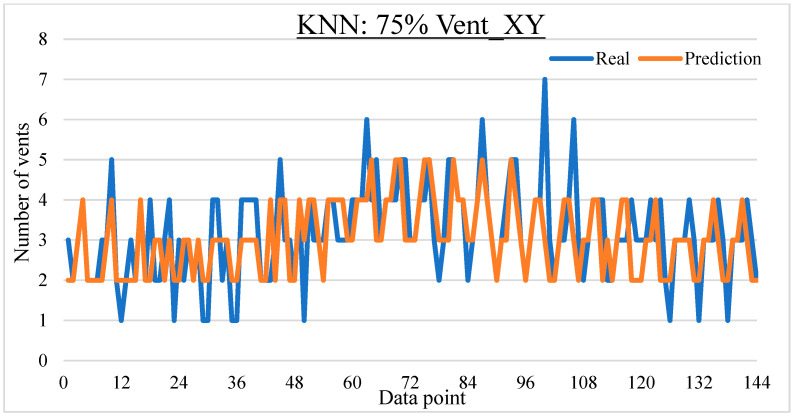
Comparison between the real and KNN predicted values for the Vent_XY dataset at 75% training data proportion.

**Figure 20:**
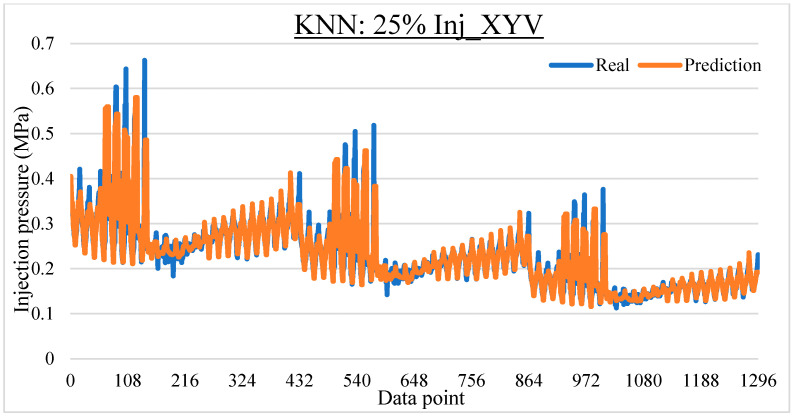
Comparison between the real and KNN predicted values for the Inj_XYV dataset at 25% training data proportion.

**Figure 21:**
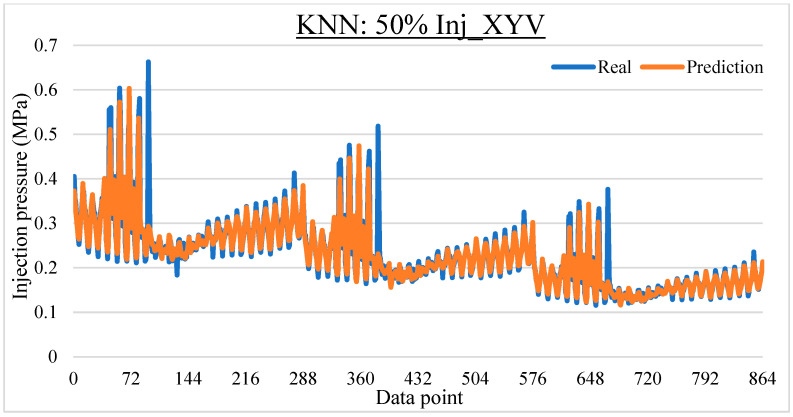
Comparison between the real and KNN predicted values for the Inj_XYV dataset at 50% training data proportion.

**Figure 22:**
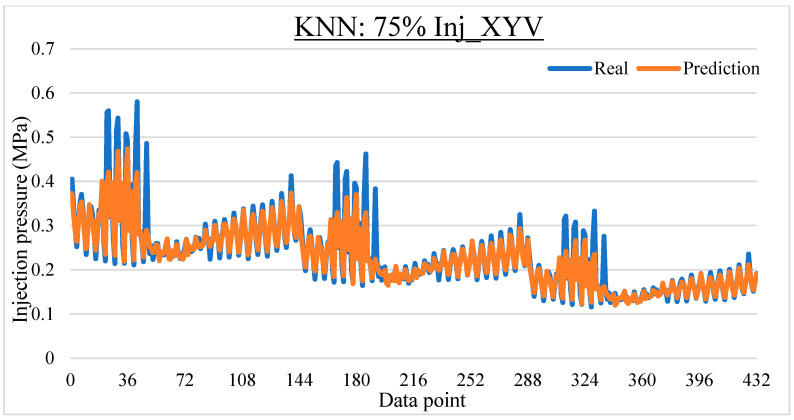
Comparison between the real and KNN predicted values for the Inj_XYV dataset at 75% training data proportion.

**Figure 23:**
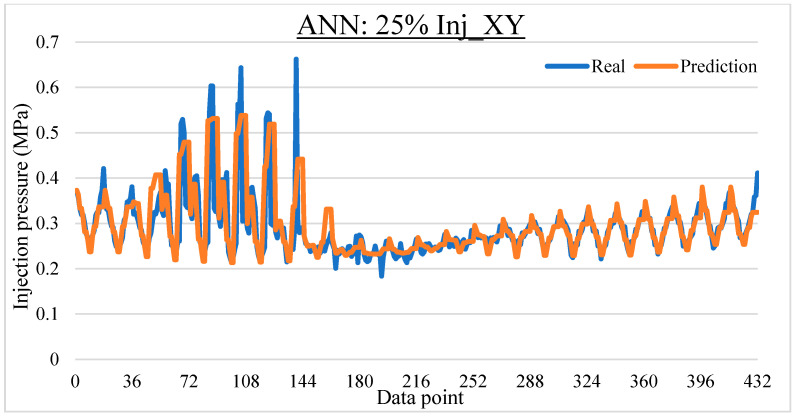
Comparison between the real and ANN predicted values for Inj_XY dataset at 25% training data proportion.

**Figure 24:**
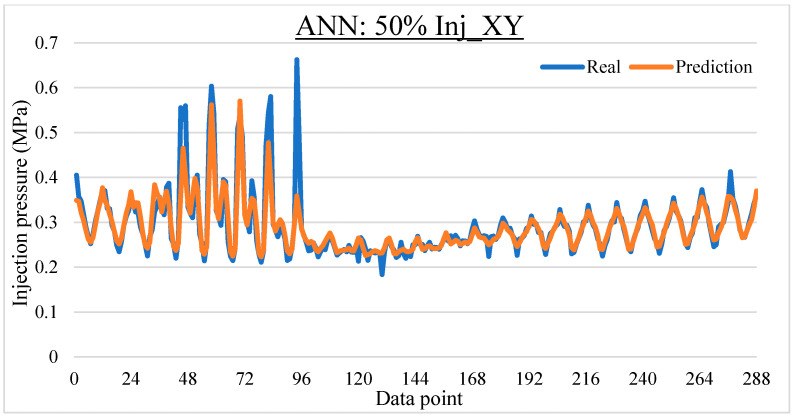
Comparison between the real and ANN predicted values for Inj_XY dataset at 50% training data proportion.

**Figure 25:**
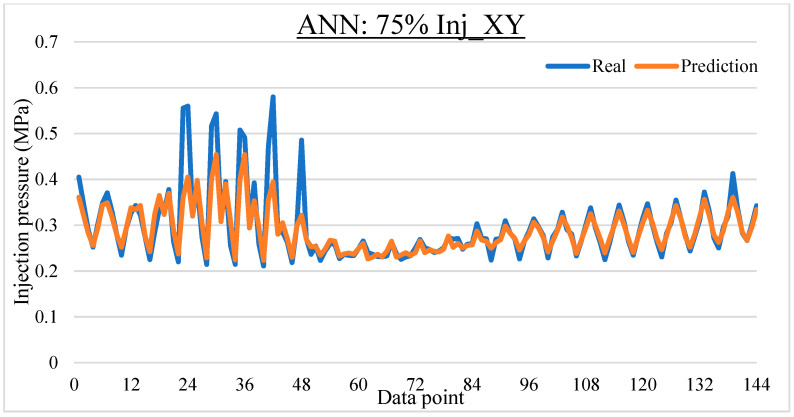
Comparison between the real and ANN predicted values for Inj_XY dataset at 75% training data proportion.

**Figure 26:**
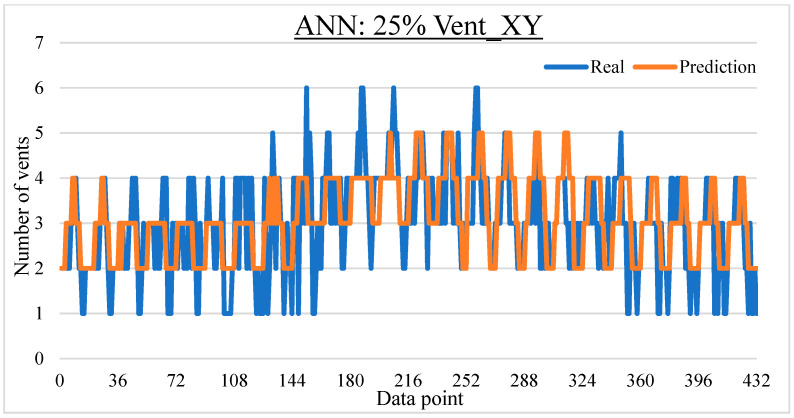
Comparison between the real and ANN predicted values for the Vent_XY dataset at 25% training data proportion.

**Figure 27:**
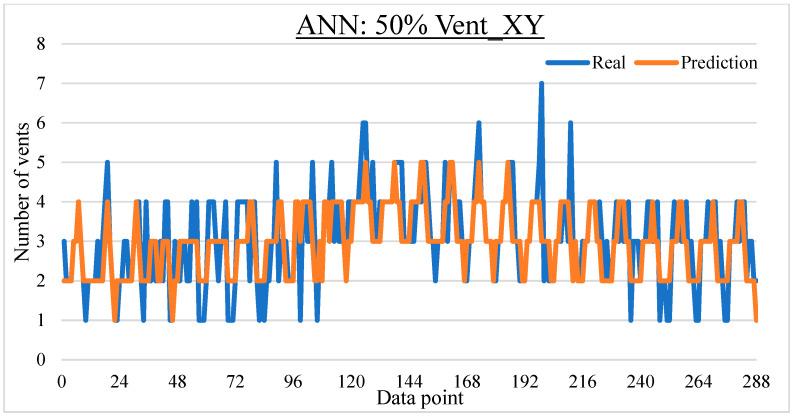
Comparison between the real and ANN predicted values for the Vent_XY dataset at 50% training data proportion.

**Figure 28:**
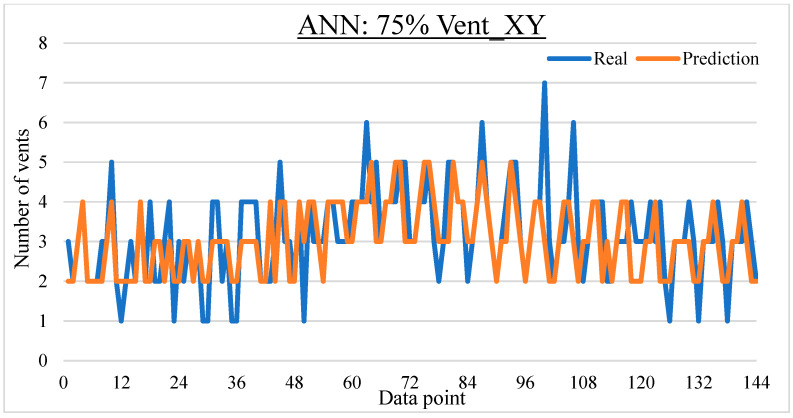
Comparison between the real and ANN predicted values for the Vent_XY dataset at 75% training data proportion.

**Figure 29:**
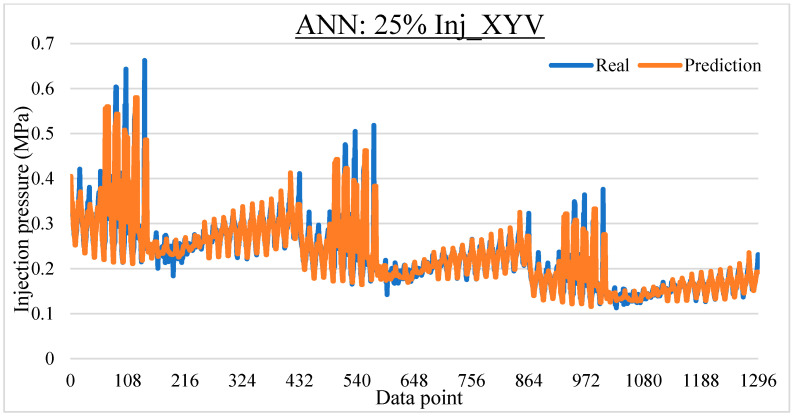
Comparison between the real and ANN predicted values for the Inj_XYV dataset at 25% training data proportion.

**Figure 30:**
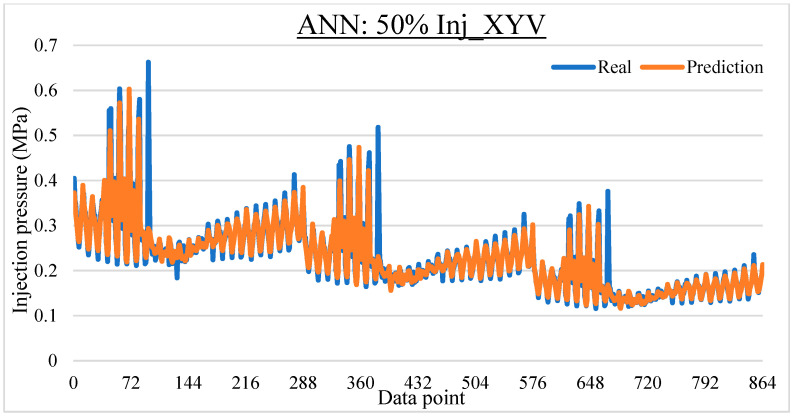
Comparison between the real and ANN predicted values for the Inj_XYV dataset at 50% training data proportion.

**Figure 31:**
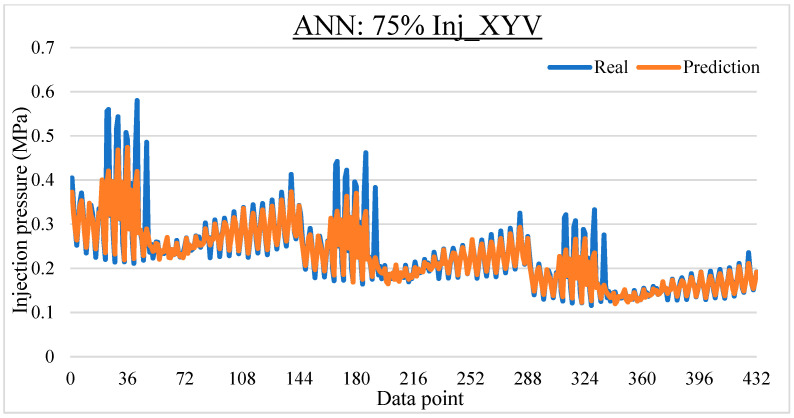
Comparison between the real and ANN predicted values for Inj_XYV dataset at 75% training data proportion.

**Table 1:** Datasets collected and their respective input/output features.

Inj_XY Dataset	Vent_XY Dataset	Inj_XYV Dataset
*x* (Input, Integer): Horizontal coordinate of gate	*x* (Input, Integer): Horizontal coordinate of gate	*x* (Input, Integer): Horizontal coordinate of gate
*y* (Input, Integer): Vertical Coordinate of gate	*y* (Input, Integer): Vertical coordinate of gate	*y* (Input, Integer): Vertical coordinate of gate
Injection pressure (Output, Double): Resultant maximum injection pressure using inputs above	Number of vents (Output, Integer): Resultant number of vents required using inputs above	Viscosity (Input, Double): Resin viscosity
		Injection pressure (Output, Double): Resultant maximum injection pressure using inputs above

**Table 2:** The corresponding n and *k* values for 50% training data proportion of each dataset.

Training Data Proportion	*n*	Recommended *k* Value
50% (Inj_XY, Vent_XY)	288	16–17
50% (Inj_XYV)	864	29–30

**Table 3:** Pseudocode of the KNN metamodel developed and investigated in this study.

***PROCEDURE***	*K-NEAREST NEIGHBOURS*
***BEGIN***	
	*Split dataset into training set and validation set*
	
	*Define k value*
***REPEAT***	
	*Calculate the distance between the query point and the training points using the Euclidean distance formula*
	
	*Add the distance and the index of the training point to an ordered collection of distance subsets*
	
	*Sort the ordered collection of distance subsets and indices by the distances in ascending order*
	
	*Pick the top k distance subsets from the sorted collection*
	
	*Get the labels of the selected k subsets*
	
	*Include the mean of k labels to the prediction dataset*
	
***UNTIL***	*All queries in the validation dataset are calculated*
	
***RETURN***	*Prediction dataset*
***END***	

**Table 4:** Compilation of the resultant prediction errors (RMSE) at various parameter levels.

Dataset Studied	No. of Hidden Neurons	Learning Rate	RMSE
Inj_XY 50% Data proportion	25	0.9	0.04184
	20	0.9	0.04794
	15	0.9	0.04788
	5	0.9	0.06042
	25	0.7	0.04656
	20	0.7	0.05075
	15	0.7	0.05377
	5	0.7	0.06047
	25	0.5	0.05038
	20	0.5	0.05038
	15	0.5	0.05042
	5	0.5	0.06054
	25	0.3	0.05050
	20	0.3	0.05067
	15	0.3	0.04869
	5	0.3	0.04841
	25	0.1	0.05390
	20	0.1	0.05267
	15	0.1	0.05262
	5	0.1	0.05561

**Table 5:** Compilation of the resultant prediction errors (RMSE) with different numbers of hidden neurons adopted.

Dataset Studied	No. of Hidden Neurons	Learning Rate	RMSE
Inj_XY 50% Data proportion	50	0.9	0.04620
	45	0.9	0.04595
	40	0.9	0.04839
	35	0.9	0.04271
	30	0.9	0.04476

**Table 6:** Pseudocode of the ANN metamodel developed and investigated in this study.

***PROCEDURE***	*ARTIFICIAL NEURAL NETWORK*
***BEGIN***	
	*Split dataset into training set and validation set*
	*Scale input values for the training set*
	*Scale the output values for ‘Vent_XY’ dataset*
	*Initialise parameter W and b*
	*Define the epoch, learning rate, and number of hidden neurons*
***REPEAT***	
	*Forward propagation*
	
	*Compute the loss using Root Mean Square Error*
	*Calculate the gradient of loss*
	*Calculate new W and b using the gradient and update the parameters*
***UNTIL***	*Max epoch has reached*
***RETURN***	*Trained model with updated parameters (W, b)*
	*Trained model is used to predict the test dataset*
	*RMSE of the model prediction and real value is presented*
***END***	

**Table 7:** Qualitative comparison between metamodels for factors influencing metamodel adoptions.

Metamodel	KNN	ANN
Interpretability	High	Low
Volume of training data required	Low	High
Ease of development and implementation	High	Moderate

**Table 8:** Computation time comparisons between the KNN and ANN metamodels.

Dataset	Data Proportion	Average Computation Time (s)	
		KNN	ANN
Inj_XY, Vent_XY	25%	91	120
	50%	122	126
	75%	158	124
Inj_XYV	25%	188	127
	50%	302	121
	75%	427	126

## Data Availability

The datasets generated during and/or analyzed during the current study are not publicly available due to the sensitive nature of the industry collaboration, but are available from the corresponding author on reasonable request.
